# *Bifidobacterium animalis* subsp. *lactis* Probio-M8 enhances chondroitin efficacy for knee osteoarthritis in postmenopausal women via the gut-joint axis

**DOI:** 10.1128/msystems.00862-25

**Published:** 2025-11-28

**Authors:** Kexin Wang, Hanbo Wang, Zhixin Zhao, Xin Shen, Jianmin Zhao, Heping Zhang

**Affiliations:** 1 Inner Mongolia Key Laboratory of Dairy Biotechnology and Engineering, Inner Mongolia Agricultural University 117454 , Hohhot, Inner Mongolia, China; 2 Key Laboratory of Dairy Products Processing, Ministry of Agriculture and Rural Affairs, Inner Mongolia Agricultural University 117454 , Hohhot, Inner Mongolia, China; 3 Key Laboratory of Dairy Biotechnology and Engineering, Ministry of Education, Inner Mongolia Agricultural University 117454 , Hohhot, Inner Mongolia, China; 4 Collaborative Innovative Center for Lactic Acid Bacteria and Fermented Dairy Products, Ministry of Education, Inner Mongolia Agricultural University 117454 , Hohhot, Inner Mongolia, China; 5 The Second Affiliated Hospital of Inner Mongolia Medical University 653268 , Hohhot, Inner Mongolia, China; 6 Department of Orthopaedics, The Affiliated Hospital of Inner Mongolia Medical University 159375 , Hohhot, Inner Mongolia, China; The University of Hong Kong, Hong Kong, Hong Kong

**Keywords:** knee osteoarthritis, probiotic adjuvant therapy, *Bifidobacterium animalis* subsp. *lactis* Probio-M8, gut microbiome, gut metabolome

## Abstract

Knee osteoarthritis (KOA) is a chronic joint disease marked by cartilage degradation and inflammation. Probiotics exhibit anti-inflammatory properties and may influence the gut-joint axis. Thus, a 4-month human trial was conducted to assess the adjunctive effects of *Bifidobacterium animalis* subsp. *lactis* Probio-M8 on KOA in postmenopausal women. Sixty-five KOA patients were randomly allocated to the probiotic group (*n* = 37; Probio-M8 and chondroitin sulfate) or placebo group (*n* = 28; placebo and chondroitin sulfate). Following a 3-month intervention, participants from both groups entered a 1-month observation without probiotic supplementation. Our findings revealed that Probio-M8 co-administration significantly reduced Western Ontario and McMaster Universities Osteoarthritis Index (WOMAC) scores at months 1, 3, and 4 compared to the placebo group (*P* < 0.001). The probiotic group showed a significant decrease in serum IFN-γ and increases in IL-4 and IL-10 (*P* < 0.05). Fecal metagenome analysis showed significant changes in the gut microbiota of the probiotic group, with increases in potentially beneficial species, including *Agathobaculum butyriciproducens*, *Bacteroides stercoris*, *B. animalis*, *Roseburia hominis*, and *Ruminococcus bromii*, while *Dorea formicigenerans* decreased (*P* < 0.05). Changes in *B. animalis* were strongly associated with WOMAC scores. The gut metabolic potential analysis showed elevated levels of N-oleoylethanolamine and decreased levels of cholesterol and hypoxanthine in probiotic receivers (*P* < 0.05). Metabolite analysis revealed post-interventional alternations in fecal prostaglandin E2, stearic acid, cholic acid, chenodeoxycholic acid, xanthine, testosterone, and serum bile acids (*P* < 0.05). Collectively, Probio-M8 enhances the effectiveness of chondroitin sulfate in KOA management through modulating the gut-joint axis, potentially via regulating multiple inflammatory pathways.

**IMPORTANCE:**

The pathogenesis of knee osteoarthritis (KOA) and its phenotypic expression have been associated with the human gut microbiota. Our study demonstrated that the co-administration of Probio-M8 with chondroitin sulfate significantly alleviates KOA symptoms. This probiotic intervention enhances therapeutic efficacy through modulation of the gut microbiota and associated metabolic pathways, reducing inflammation and improving clinical outcomes. Our results underscore the potential of probiotic-driven therapies as an adjunctive treatment strategy and underscore the importance of the gut-joint axis in KOA management.

## INTRODUCTION

Osteoarthritis (OA) is a degenerative joint disease marked by cellular inflammatory responses and subsequent degenerative changes in the joint cartilage (1). In 2019, it was estimated that approximately 528 million individuals worldwide were affected by OA (2). Knee OA (KOA), the predominant OA phenotype, engenders persistent joint pain and progressive functional decline, markedly compromising range of motion and health-related quality of life (3). In China, the incidence of KOA in 2019 was 305 per 100,000 individuals among males and 500 per 100,000 individuals among females (4). Notably, the risk of KOA increases rapidly among females aged between 55 and 75, particularly among postmenopausal women (5). The etiology of KOA in this demographic is multifactorial, involving diet, obesity, genetic predisposition, joint-specific factors, and aging-associated estrogen deficiency (6). Accumulating evidence highlights the importance of gut microbiota in KOA pathogenesis (7, 8). The gut microbiota significantly influences host physiological processes, including inflammatory responses (9). For example, fecal microbiota transplantation from KOA patients induces heightened inflammation and increased intestinal permeability in germ-free mice (10). Additionally, KOA-related inflammatory responses can be modulated through host-microbial interactions or probiotic interventions (11, 12). This underscores the significance of the gut-joint axis in KOA management, suggesting that targeting gut microbiota could provide new therapeutic avenues for mitigating KOA symptoms.

Probiotics are live microorganisms that bring beneficial effects on human health when consumed in sufficient amounts (13). Emerging evidence has demonstrated that probiotics can modulate inflammatory responses and promote bone health (14–16). Preclinical evidence highlights the protective and therapeutic effects of specific probiotic strains on KOA. For instance, *Lactobacillus plantarum* GKD7 has been shown to improve subchondral bone health, maintain cartilage architecture, and reduce synovial inflammation in KOA models (17). Similarly, strains such as *Lactobacillus rhamnosus* LR-2 (18), *Lactobacillus delbrueckii* subsp. *lactis* 557 (19), *Lactobacillus acidophilus* LA-1 (20), and *L. acidophilus* ATCC 4356 (21) have demonstrated efficacy in protecting against cartilage damage and inflammation. Conversely, the application of probiotics in managing KOA has produced inconsistent results in human trials. While two trials have reported significant reductions in Western Ontario and McMaster Universities Osteoarthritis Index (WOMAC) scores among patients (22, 23), other studies have failed to show a decrease in the WOMAC OA index score (24, 25). This variability in therapeutic efficacy may be attributed to strain-specific effects and inter-individual differences. Moreover, mechanisms by which probiotic-based gut microbiota and metabolite change influence KOA symptoms remain to be fully elucidated.

The probiotic strain *Bifidobacterium animalis* subsp. *lactis* Probio-M8 (Probio-M8) was originally isolated from the breast milk of a healthy woman in 2017 (26). This strain has been characterized for its ability to mitigate inflammation and oxidative stress, while enhancing the abundance of beneficial intestinal bacteria, thereby providing a protective effect for the host (27–29). Additionally, our prior clinical investigation demonstrated that the administration of Probio-M8 in conjunction with conventional pharmacotherapy may augment bone metabolism in postmenopausal osteoporosis patients through modulating the gut microbiota (30). This study described a 4-month human trial examining the alleviative efficacy of Probio-M8 in postmenopausal women with KOA. The study aimed to assess the alleviative effects of Probio-M8 on KOA symptoms and to elucidate the underlying mechanisms by examining alternations in clinical parameters, gut microbiota, and gut metabolites. The results of this investigation are expected to yield valuable understanding regarding the pathogenesis and therapeutic approaches for KOA, as well as the potential mechanisms of innovative disease management and the utilization of probiotics-driven interventions.

## MATERIALS AND METHODS

### Study design and subject recruitment

This 4-month longitudinal study was conducted at the Affiliated Hospital of Inner Mongolia Medical University from June 2021 to February 2022. Ethical approval for the study protocol was obtained from the Ethics Committee of the Affiliated Hospital of Inner Mongolia Medical University (project number KY2021018). Sixty-five postmenopausal women aged 50–70 years with a clinical diagnosis of KOA were recruited. The diagnosis was made according to China’s national guidelines for the diagnosis and treatment of OA (2018) as stated by the Joint Surgery Group, Orthopedics Branch, Chinese Medical Association. Detailed diagnostic criteria are tabulated in Table S1.

Inclusion criteria were as follows: (i) postmenopausal women aged 50–70 years and (ii) clinical diagnosis of KOA. Exclusion criteria included (i) use of probiotics or prebiotics within the last 2 weeks; (ii) known allergy to lactic acid bacteria or their products; (iii) presence of cardiovascular diseases, renal insufficiency, malignant tumors, infectious diseases, or digestive tract diseases; (iv) history of abdominal surgery; (v) requirement for antibiotic treatment during the study period; and (vi) mental conditions compromising the ability to provide informed consent.

After the initial screening, which excluded 12 patients, 68 eligible participants were randomly allocated to the probiotic group (*n* = 38) or the placebo group (*n* = 30). One patient in the probiotic group and two patients in the placebo group withdrew. The final cohorts comprised 37 patients in the probiotic group and 28 in the placebo group. Following a 3-month intervention period, participants from both groups underwent a 1-month observation period without probiotic supplementation for evaluating the sustained impact of Probio-M8. During the 1-month observation period, the number of participants retained for assessment at month 4 was reduced to 33 in the probiotic group and 25 in the placebo group. Group allocation was blinded to the participants throughout the study.

The probiotic group consumed 2 g of probiotic powder containing at least 5 × 10^10^ CFU of Probio-M8 daily for 3 months. The placebo group consumed an identical-appearing placebo powder. The probiotic and placebo materials were both prepared and packaged by JinHua YinHe Biological Technology Co., Ltd., Zhejiang, China, and stored at 4°C. Throughout the entire experimental period, all participants also received a daily dose of chondroitin sulfate (900 mg) provided by Suzhong Pharmaceutical Group Co., Ltd, Jiangsu, China. Patients were instructed not to use any additional probiotics or prebiotics during the study period.

### Clinical assessment and sample collection

The primary outcome measure in this study was the change in the WOMAC scores, a validated and widely utilized instrument for evaluating the severity of OA symptoms (31). The WOMAC index comprises three subscales: pain (five items), stiffness (two items), and physical function (17 items). Each item is scored from 0 to 4, with higher scores implying greater severity of symptoms. The total possible scores for the subscales are 0–20 for pain, 0–8 for stiffness, and 0–68 for physical function, respectively.

Control indicators for participants, including WOMAC scores, and levels of inflammatory cytokines (interleukin (IL)-2, IL-4, IL-6, IL-10, IL-17A, TNF-α, and IFN-γ), were assessed at months 0, 1, 3, and 4. Levels of inflammatory cytokines were measured using an automated analyzer (DXA5000, Beckman Coulter, Inc., CA, USA) following the manufacturer’s protocols. At baseline, no significant differences were detected between the Probio-M8 and placebo groups with respect to age, WOMAC scores, or inflammatory cytokine levels (*P* > 0.05; Table S1). Fecal and blood samples were collected simultaneously (months 0, 1, 3, and 4) for further analysis. Due to the discontinuation of fecal and blood sample donations, only a subset of samples was available for subsequent analyses (Fig. S1).

Fecal samples intended for microbial analysis were collected using the Longsee stool storage kit (Guangdong Longsee Biomedical Co., Ltd, Guangzhou, China). For metabolic analysis, fecal samples were collected using sterile techniques. All fecal samples were stored in standard sampling tubes at −80℃ until required for analysis.

Blood samples were collected in sterile tubes containing EDTA and promptly processed by centrifuging to isolate the serum. The resulting serum samples were stored at −80°C until required for analysis.

### DNA extraction and metagenomic sequencing

Fecal DNA extraction was conducted by the QIAamp DNA Stool Mini Kit (Qiagen GmbH, Hilden, Germany). The quality and integrity of DNA were evaluated using 0.8% agarose gel electrophoresis and quantified using a fluorometer (Qubit 2.0, Life Technologies, CA, USA). DNA samples meeting the quality criteria were utilized for library preparation with the NEBNext Ultra DNA Library Prep Kit (New England Biolabs, Ipswich, MA, USA). The paired-end reads were generated through sequencing on the Illumina NovaSeq platform (Illumina Novaseq 6000; Illumina Inc., San Diego, CA, USA). Sequencing services were provided by Tianjin Novogene Technology Co., Ltd. (Tianjin, China).

### Bioinformatic analysis pipeline

Quality control of the metagenomic sequencing data were conducted via KneadData (version 0.7.5; http://huttenhower.sph.harvard.edu/kneaddata). Specifically, reads <60 nt were removed using Trimmomatic, and sequences were depleted to enrich for microbial reads (32). Subsequently, the high-quality reads were analyzed using the HUMAnN2 software suite (33). Microbial taxonomic profiling was conducted using MetaPhlAn3, which calculates the relative abundance of gut microbiota (34). Pan-genome nucleotide alignment at the species level was performed using Bowtie 2 software in conjunction with the ChocoPhlAn database (version 3.0.1) (35). Unaligned sequences were further processed with Diamond software to align against the UniRef 90 database and generate putative protein sequences (36). These protein sequences were then analyzed using the core HUMAnN2 algorithm to determine the pathway abundance of the microbiome. MelonnPan pipeline was employed to predict gut metabolites based on the microbial community composition (37).

### Fecal metabolite extraction and analysis

A fecal sample aliquot was added to a methanol solution (600 µL) with 2-chlorophenylalanine (4 ppm) and mixed for 30 seconds and then subjected to mechanical grinding, ultrasonication, centrifugation, and filtration prior to analysis. The fecal metabolome was analyzed by an LC20 ultra-performance liquid chromatography system (Shimadzu Corporation, Kyoto, Japan) coupled with a TripleTOF-6600 mass spectrometer (AB Sciex Pte. Ltd., Framingham, MA, USA). The analysis was conducted by MetWare Biotechnology Co., Ltd. (Wuhan, China).

### Quantification of blood metabolite

Blood samples were analyzed for prostaglandin E2, lysophosphatidylcholines (lysoPCs), phosphatidylcholines (PCs), and omega-6 polyunsaturated fatty acids (n-6 PUFAs) using enzyme-linked immunosorbent assay kits (Meimian Industrial Co., Ltd., Jiangsu, China). The assays were conducted following the manufacturer’s protocols to ensure standardized and reproducible results.

Bile acids were extracted from blood samples (50 µL) using 200 µL of methanol/acetonitrile (vol/vol = 2:8) and then evaporated to dryness. The residues were analyzed by an LC-MS/MS system (UHPLC ExionLC AD, AB Sciex Pte. Ltd., Framingham, MA, USA; MS Applied Biosystems 6500 Triple Quadrupole, AB Sciex Pte. Ltd., Framingham, MA, USA).

### Statistical analysis and visualization

**Fig 1:**
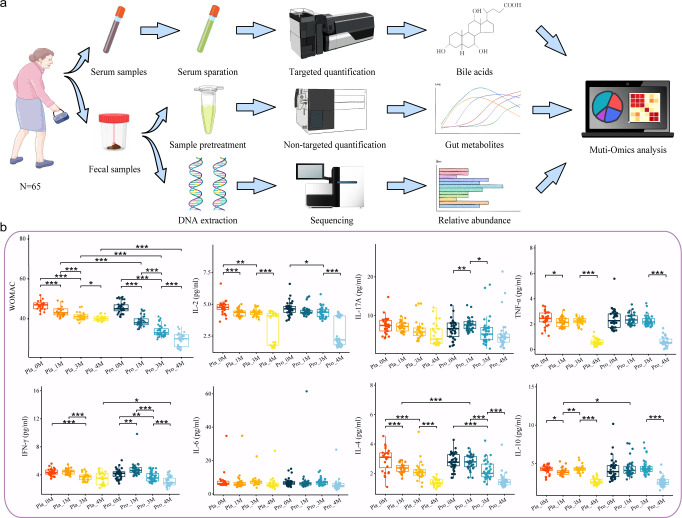
Multi-omics analysis workflow and clinical parameter assessment in KOA patients. (**a**) Multi-omics analysis workflow depicts the comprehensive process of sampling, preparation, detection, and analysis. (**b**) Statistical differences in WOMAC scores and inflammatory cytokines (IL-2, IL-17A, TNF-α, IFN-γ, IL-6, IL-4, and IL-10) within and between the Probio-M8 group (Pro) and the placebo group (Pla) at various time points: baseline (0M), after 1 month of intervention (1M), after 3 months of intervention (3M), and after an additional 1-month follow-up period (4M). Statistical significance was evaluated by the Wilcoxon test (* *P* < 0.05, ** *P* < 0.01, *** *P* < 0.001).

Statistical analyses were performed by R software (version 4.1.2). The Shannon-Wiener diversity index and principal coordinates analysis (PCoA) were analyzed using R packages such as ggplot2, ggpubr, and vegan. The Wilcoxon test was employed to evaluate inter-group and intra-group differences in WOMAC scores, inflammatory cytokines, fecal microbiota profiles, gut metabolic pathways, predicted bioactive metabolites, and blood metabolites. Multivariate relationships between gut microbiome and clinical parameters were explored using MaAslin2 (38). All statistical visualizations were generated using R and subsequently refined for publication using Adobe Illustrator. The icons, which have undergone minor modifications, were sourced from Bioicons (https://bioicons.com/) and are licensed under the Creative Commons Attribution 4.0 International License (https://creativecommons.org/licenses/by/4.0/). The workflow of the multi-omics analysis is illustrated in Fig. 1a.

STORMS checklist is available at https://doi.org/10.5281/zenodo.17045320.

## RESULTS

### Probio-M8 improved KOA-related symptoms

To assess effects of Probio-M8 in alleviating symptoms associated with KOA, a comprehensive evaluation of clinical parameters was conducted at months 0, 1, 3, and during a follow-up at month 4. These parameters included WOMAC scores and serum levels of inflammatory cytokines (IL-2, IL-4, IL-6, IL-10, IL-17A, TNF-α, and IFN-γ). At baseline (month 0), no significant differences were detected between the Probio-M8 and placebo groups in these indicators. During the study period, both groups demonstrated significant reductions in WOMAC scores (*P* < 0.05; Fig. 1b). However, the Probio-M8 group exhibited significantly lower WOMAC scores compared to the placebo group at months 1, 3, and 4 (*P* < 0.001; Fig. 1b). During the follow-up period, WOMAC scores in the Probio-M8 group continued to decline significantly compared to those in the placebo group (*P* < 0.001 for Probio-M8 group, *P* = 0.029 for placebo group, from month 3 to month 4; Fig. 1b).

Levels of pro-inflammatory cytokines IL-2, IL-17A, and TNF-α decreased in both groups over the study period, with no significant inter-group differences (*P* > 0.05; Fig. 1b). Notably, the IFN-γ level at month 4 was significantly lower in the Probio-M8 group compared to the placebo group (*P* = 0.048; Fig. 1b). Conversely, no significant alterations in IL-6 levels were detected within or between groups throughout the study (*P* > 0.05; Fig. 1b). The Probio-M8 group demonstrated significantly elevated levels of the anti-inflammatory cytokines IL-4 and IL-10 in comparison to the placebo group at month 1 (*P* < 0.001 and *P* = 0.023, respectively; Fig. 1b). These results indicated that the adjunctive treatment with Probio-M8 significantly improved KOA-related symptoms and inflammatory status compared to chondroitin sulfate treatment alone.

### Probio-M8 modified gut microbiota composition

**Fig 2:**
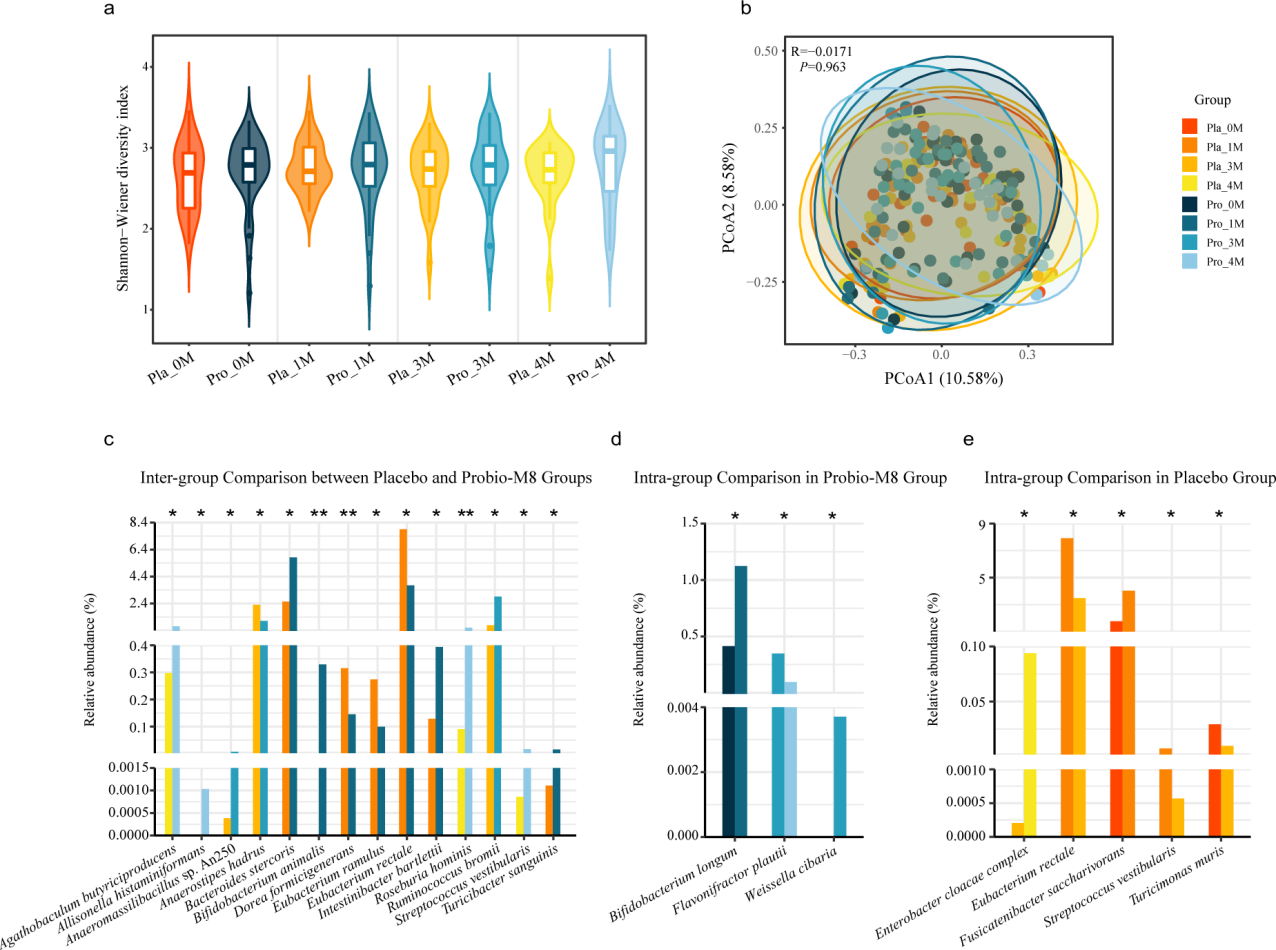
Microbial diversity and differential abundance in fecal metagenome data. (**a**) Shannon-Wiener diversity index was calculated for the Probio-M8 (Pro) and placebo (Pla) groups at months 0 (0M), 1 (1M), 3 (3M), and 4 (4M). No significant differences were observed between the two groups, as determined by the Wilcoxon test. (**b**) PCoA plot, based on Bray-Curtis distance, illustrates the clustering patterns of the fecal microbiota. No significant differences in clustering patterns were detected between the groups or over time. (**c**) Inter-group comparisons of species abundance between the Probio-M8 and placebo groups at the same time points. All the shown species were not significantly different between groups at baseline. (**d–e**) Intra-group comparisons of species abundance within the Probio-M8 group (**d**) and the placebo group (**e**) across time points. Statistical significance was evaluated by the Wilcoxon test (* *P* < 0.05, ** *P* < 0.01, *** *P* < 0.001).

No significant differences in alpha diversity, as assessed by the Shannon-Wiener diversity index, were observed between the Probio-M8 and placebo groups at any time point (*P* > 0.05; Fig. 2a). Similarly, beta diversity analysis, evaluated by PCoA with Bray-Curtis distances, did not reveal significant changes in clustering patterns based on group or time (*P* > 0.05; Fig. 2b). These findings indicate that Probio-M8 did not induce significant changes in the overall diversity and structure of the gut metagenome.

To further investigate alternations in the gut microbiome during the intervention, we conducted a finer taxonomic analysis. At baseline, no significant differences were noted between the Probio-M8 group and the placebo group (*P* > 0.05). Inter-group comparisons between species abundance of Probio-M8 and placebo groups at the same time point exhibited 14 significantly differential species (*P* < 0.05, Fig. 2c). At month 1, the Probio-M8 group showed a significantly higher abundance of *Bacteroides stercoris*, *B. animalis*, *Intestinibacter bartlettii*, and *Turicibacter sanguinis* compared to the placebo group, while *Dorea formicigenerans*, *Eubacterium rectale*, and *Eubacterium ramulus* exhibited an inverse abundance trend. At month 3, the Probio-M8 group displayed significantly higher abundance levels compared to the placebo group for species such as *Anaeromassilibacillus* sp. An250 and *Ruminococcus bromii*, while *Anaerostipes hadrus* displayed an opposite abundance trend. Four probiotic-responsive species, including *Agathobaculum butyriciproducens*, *Allisonella histaminiformans*, *Roseburia hominis*, and *Streptococcus vestibularis*, increased significantly after the follow-up period.

Intra-group comparisons of species abundance in the Probio-M8 group at different time points revealed that *Bifidobacterium longum* and *Weissella cibaria* elevated significantly, whereas *Flavonifractor plautii* declined significantly (*P* < 0.05, Fig. 2d). Intra-group analyses within the placebo group revealed significant temporal changes: the abundance of *E. rectale*, *S. vestibularis*, and *T. sanguinis* declined significantly during/after the intervention, whereas *Fusicatenibacter saccharivorans* increased significantly during the intake period (*P* < 0.05, Fig. 2e). The abundance of *Enterobacter cloacae complex* grew significantly after the follow-up period (*P* < 0.05, Fig. 2e). These findings indicated that, despite the absence of drastic alternations in the overall gut microbiome, the abundance of specific species underwent significant alterations during the study period.

### Probio-M8 regulated gut metabolic pathways and predicted bioactive compounds

To further investigate the impact of Probio-M8 intervention on the gut microbiome, we performed an in-depth analysis of metabolic pathways encoded in subjects’ gut microbiota using the MetaCyc and KEGG databases. This analysis identified 36 pathways that were responsive to the intervention. These pathways did not show significant differences between groups at baseline but showed significant differential abundance either within or between groups throughout the study.

**Fig 3:**
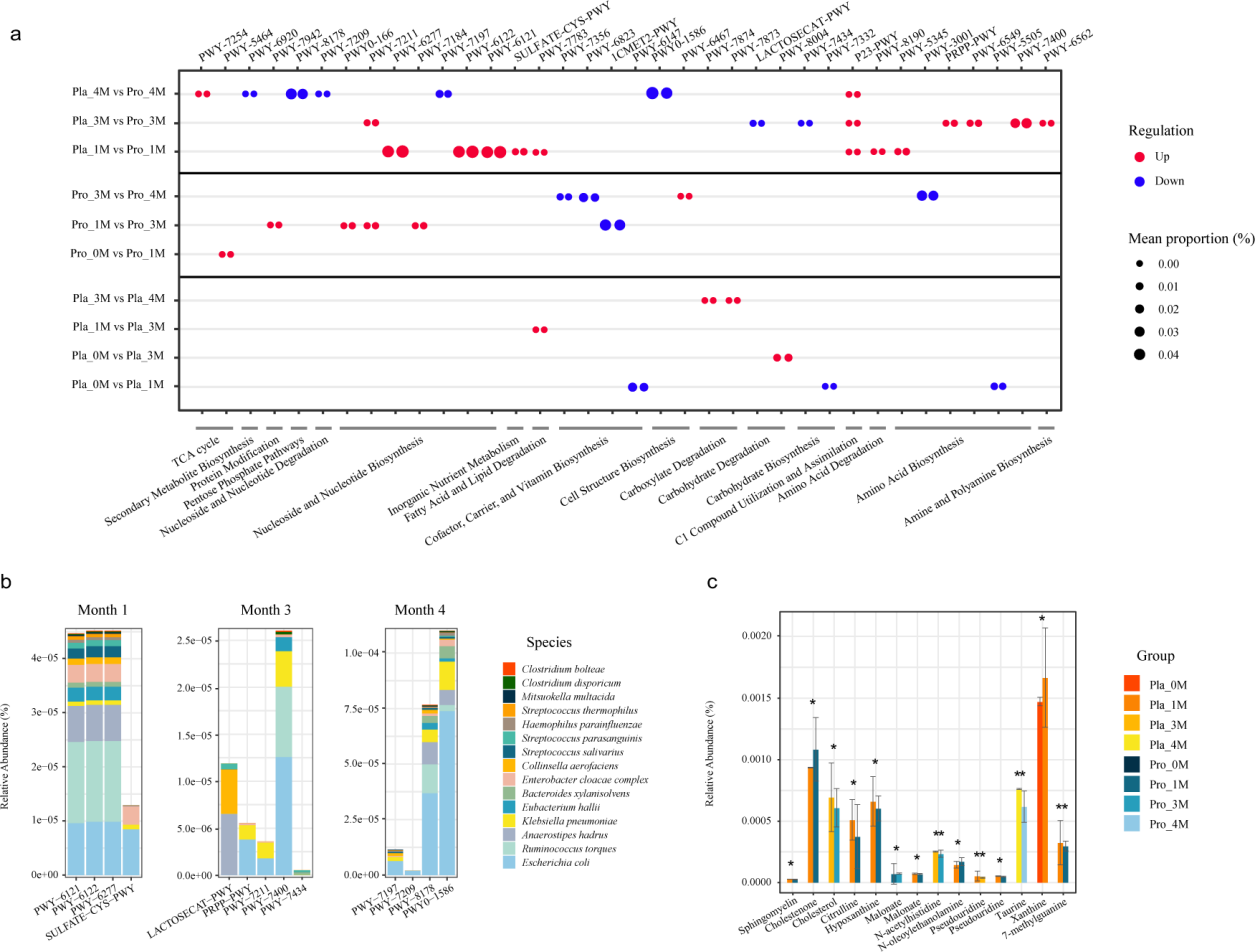
Comparative analysis of gut microbiota-encoded metabolic pathways and predicted bioactive compounds. (**a**) Comparative visualization of gut metabolic pathways in the two groups. Red and blue bubbles denote pathways with higher or lower mean proportions in the subgroup following “vs” compared to the preceding subgroup, respectively. (**b**) Contribution of species to 13 significantly differential metabolic pathways of the fecal microbiome across all patients at months 1, 3, and 4. (**c**) Bar graph depicting significant differences in predicted bioactive metabolites between the two subgroups. No significant differences were detected in gut metabolic pathways and predicted metabolites between groups at baseline. Statistical significance was evaluated by the Wilcoxon test (* *P* < 0.05, ** *P* < 0.01, *** *P* < 0.001).

Between the two groups, 21 gut microbiome-encoded metabolic pathways showed significant changes throughout the study (*P* < 0.05, Fig. 3a). Seven pathways were significantly less abundant in the Probio-M8 group compared to the placebo group (PWY-6920, PWY-8178, PWY-7209, PWY-7197, PWY0-1586, LACTOSECAT-PWY, and PWY-7434 pathways), primarily involved in nucleoside and nucleotide metabolism and carbohydrate metabolism. Conversely, the remaining 14 pathways were significantly more abundant in the Probio-M8 group than in the placebo group, including TCA cycle (PWY-7254 pathway), nucleoside and nucleotide biosynthesis (PWY-7211, PWY-6277, PWY-6122, and PWY-6121 pathways), inorganic nutrient metabolism (SULFATE-CYS-PWY pathway), fatty acid and lipid degradation (PWY-7783 pathway), C1 compound utilization and assimilation (P23-PWY pathway), amino acid metabolism (PWY-8190, PWY-5345, PRPP-PWY, PWY-6549, and PWY-7400 pathways), and amine and polyamine biosynthesis (PWY-6562 pathway).

In the Probio-M8 group, the fecal metagenome demonstrated a higher proportion of probiotic-responsive pathways, particularly those involved in tricarboxylic acid cycle (TCA cycle, PWY-5464 pathway), protein modification (PWY-7942 pathway), and nucleoside and nucleotide biosynthesis (PWY0-166, PWY-7211, and PWY-7184 pathways) during/after the intervention period (*P* < 0.05, Fig. 3a). Conversely, pathways primarily associated with cofactor, carrier, and vitamin biosynthesis (PWY-7356, PWY-6823, and 1CMET2-PWY pathways) showed a decreased proportion over time. For the placebo group, three pathways associated with cofactor, carrier, and vitamin biosynthesis (PWY-6147 pathway), carbohydrate biosynthesis (PWY-7332 pathway), and amino acid biosynthesis (PWY-5505 pathway) were observed to decrease after one month of the trial’s commencement, while pathways mainly associated with carboxylate degradation (PWY-7873 and PWY-7874 pathways) and carbohydrate degradation (PWY-8004 pathway) increased over time (*P* < 0.05, Fig. 3a).

We further explored 13 significantly differential metabolic pathways with the highest proportion between the two groups and then statistically analyzed the contribution of the gut microbiota to these pathways in all patients at different time points (*P* < 0.05; Fig. 3b). At month 1, 5-aminoimidazole ribonucleotide biosynthesis (PWY-6121, PWY-6122, and PWY-6277 pathways) was primarily dominated by *Ruminococcus torques*, *Escherichia coli*, *A. hadrus*, *E. cloacae* complex, and *Eubacterium hallii*. Cysteine biosynthesis (SULFATE-CYS-PWY pathway) mainly belonged to *E. coli* and *E. cloacae* complex. After the intake period (month 3), histidine biosynthesis (PRPP-PWY pathway) and superpathway of pyrimidine deoxyribonucleotides *de novo* biosynthesis (PWY-7211 pathway) were both linked to *E. coli*, *Klebsiella pneumoniae*, and *E. cloacae* complex. L-arginine biosynthesis (PWY-7400 pathway) was primarily attributed to *E. coli*, *R. torques*, *K. pneumoniae*, *E. hallii*, and *E. cloacae* complex. After the follow-up period (month 4), pyrimidine deoxyribonucleotide phosphorylation (PWY-7197 pathway) primarily belonged to *E. coli*, *K. pneumoniae*, and *E. cloacae* complex. Pentose phosphate pathway (non-oxidative branch) II (PWY-8178 pathway) was mainly associated with *E. coli*, *R. torques*, *A. hadrus*, *K. pneumoniae*, *Bacteroides xylanisolvens*, *E. hallii*, *Collinsella aerofaciens*, and *E. cloacae* complex.

The prediction of gut bioactive compounds was carried out using MelonnPan, and we identified 12 metabolic profiles with significant differences between subgroups (*P* < 0.05; Fig. 3c). The Probio-M8 group exhibited significantly higher levels of N-oleoylethanolamine and significantly lower levels of sphingomyelin, cholesterol, and hypoxanthine at month 1 or month 3 compared to the placebo group. These findings demonstrated that Probio-M8-induced alterations in the gut microbiome led to specific modifications in predicted metabolites and related pathways.

### Probio-M8 modulated fecal metabolites

To further elucidate changes in the fecal metabolic composition, we applied variable importance in projection (VIP) more than one to identify significant differences between the Probio-M8 and placebo groups at months 1, 3, and 4. This analysis revealed 1,123 differentially abundant metabolites. Notably, the Probio-M8 group exhibited significantly lower levels of several key fecal metabolites compared to the placebo group. These included prostaglandin E2, two saturated fatty acids (heptadecanoic acid and stearic acid), and three bile acids (chenodeoxycholic acid, cholic acid, and isohyodeoxycholic acid). Similarly, significantly reduced levels of ethenodeoxyadenosine and xanthine were observed in the Probio-M8 group at months 1 and 3. Additionally, the Probio-M8 group showed enrichment in testosterone and nicotinic acid adenine dinucleotide (NAAD) compared to the placebo group during/after the intervention. These results suggest that the consumption of Probio-M8 with conventional drugs induces specific alterations in gut metabolites of patients with KOA, potentially influencing disease-related metabolic pathways.

### Probio-M8 modulated serum bile acids

To investigate the interrelationship between gut metabolism and blood metabolism, several serum metabolites were detected throughout the study. Prostaglandin E2 is a key mediator of joint pain, inflammatory response, and cartilage matrix degradation and also a conversion product of n-6 PUFAs and the transformation of PC to lysoPC. No significant differences were detected between the Probio-M8 and placebo groups in serum levels of Prostaglandin E2 or n-6 PUFAs (*P* > 0.1, Fig. S2). However, the ratio of lysoPC to PC, a novel biomarker for predicting advanced KOA, trended lower in the Probio-M8 group compared to the placebo group at months 3 and 4, although these differences did not show statistical significance (*P* = 0.098 at month 3, *P* = 0.067 at month 4; Fig. S2).

**Fig 4:**
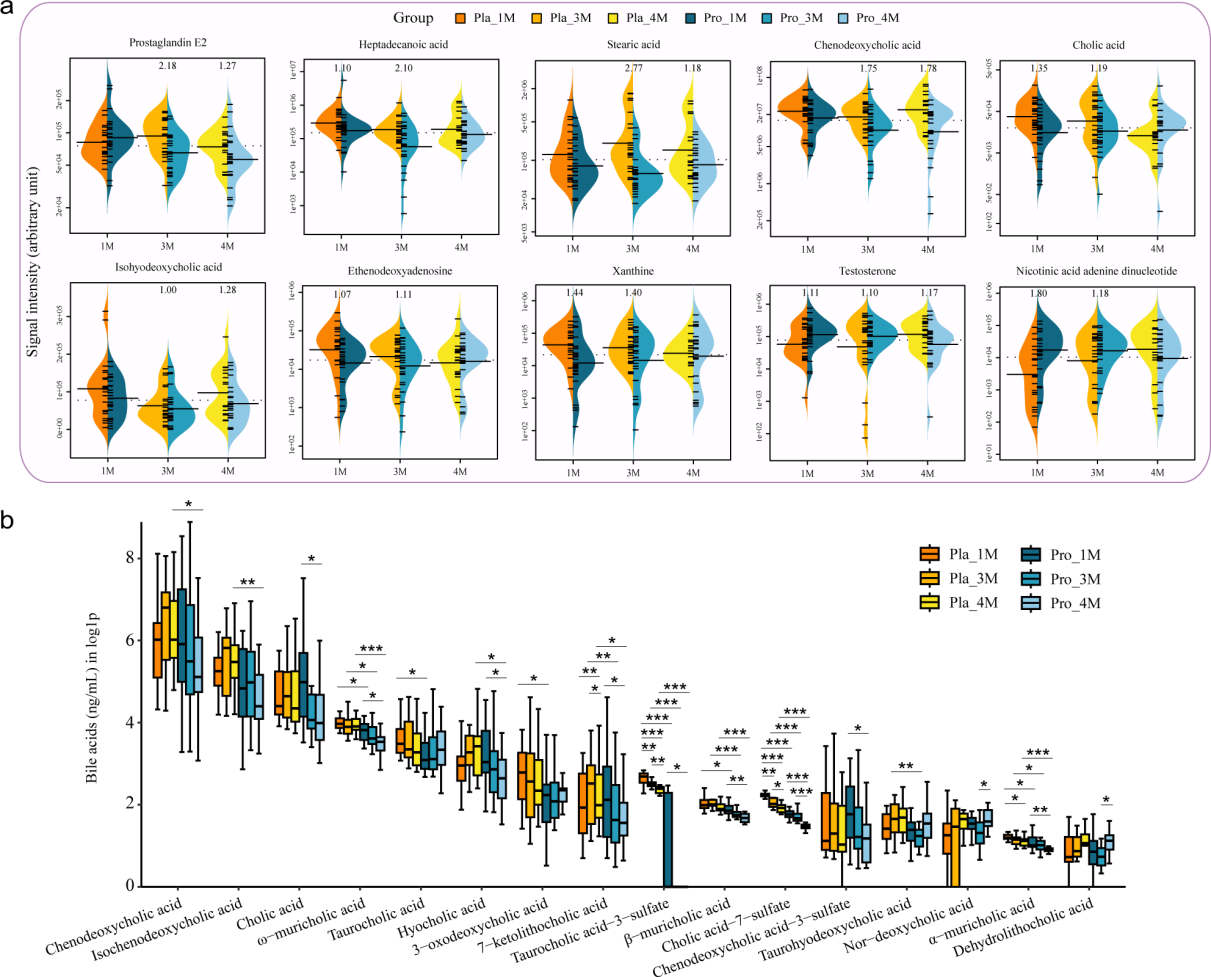
Changes in fecal metabolites and serum bile acids. (**a**) Bean plots depicting alterations in fecal levels of 10 metabolites that responded to probiotic treatment. At baseline, no significant differences were detected in these metabolites between the two groups. Subsequent statistical analysis was conducted using VIP score >1 to identify significant differences between subgroups, with VIP scores denoting significance levels. (**b**) A bar graph illustrates the levels of serum bile acids that responded to the Probio-M8 adjuvant treatment. The ordinate displays bile acid concentrations transformed via the log1p function, which computes ln(1 + x) to preserve numerical precision at low values. Statistical significance was evaluated by the Wilcoxon test (* *P* < 0.05, ** *P* < 0.01, *** *P* < 0.001).

Also, we performed a comprehensive analysis of 36 serum bile acids to investigate the relationship between gut and serum bile acids. The analysis revealed significant differences in 16 serum bile acids between the subgroups (*P* < 0.05, Fig. 4b). Notably, five bile acids (α-muricholic acid, β-muricholic acid, ω-muricholic acid, cholic acid-7-sulfate, and taurocholic acid-3-sulfate) exhibited significantly lower levels in the Probio-M8 group compared to the placebo group at months 1, 3, and 4 (*P* < 0.05). Additionally, chenodeoxycholic acid and cholic acid showed lower signal intensity in the Probio-M8 group compared to the placebo group at month 4 (*P* = 0.033 for chenodeoxycholic acid, *P* = 0.093 for cholic acid). Furthermore, a significant decrease in cholic acid levels was found within the Probio-M8 group from month 1 to month 4 (*P* = 0.024). These findings suggest that adjunctive Probio-M8 therapy modulates bile acid metabolism in patients with KOA, potentially facilitating a link between gut and serum bile acids, which could have implications for disease management.

### Correlations among microbiome, metabolome, and clinical parameters

**Fig 5:**
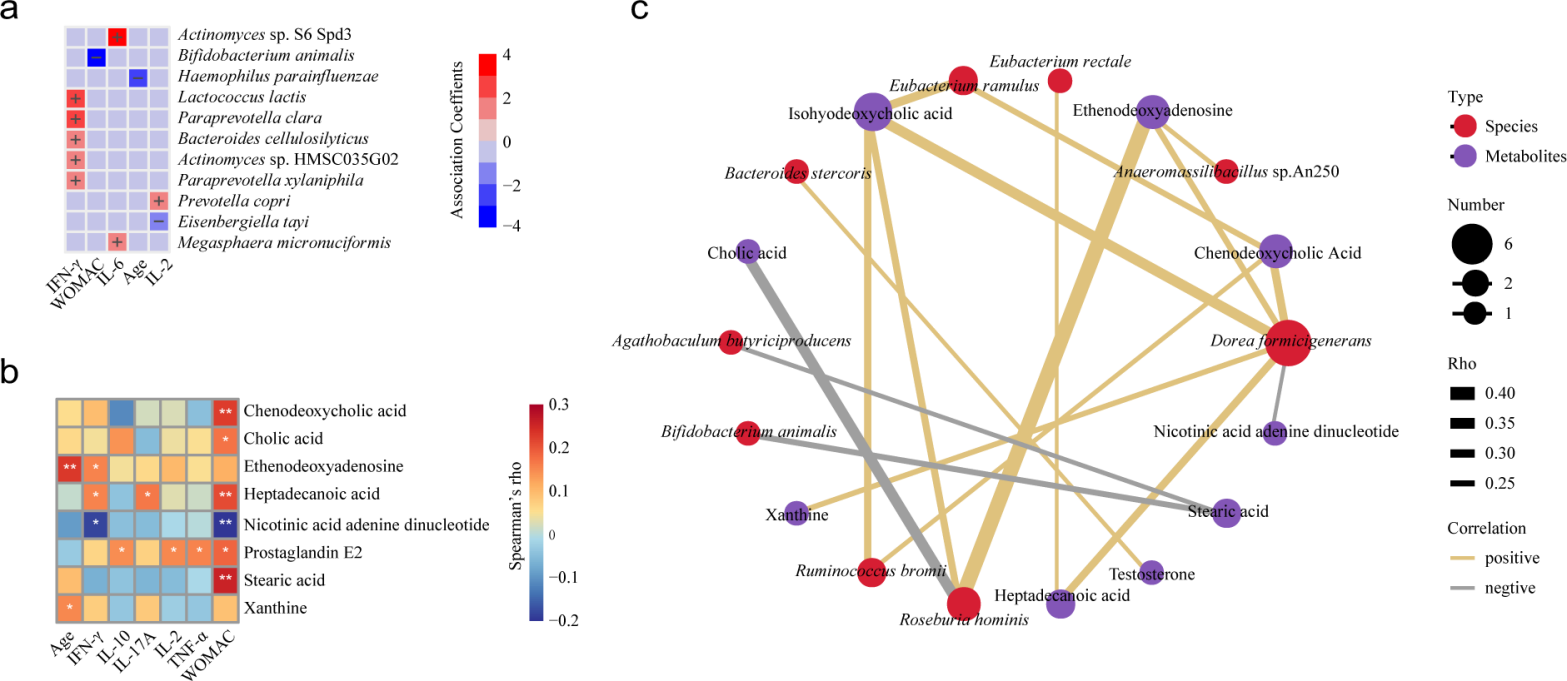
Multivariable associations and correlations between multiple parameters. (**a**) A correlation matrix illustrating multivariable association analysis between species-level microbial abundance and disease indicators. In this matrix, red hues signify positive associations, whereas blue hues denote negative associations (*P* < 0.05). (**b**) Correlation heatmap depicting the relationship between the fecal metabolome and clinical parameters in the two groups. Heatmap based on Spearman’s rank correlation; only features showing moderate-to-strong associations (|Rho| > 0.15, *P* < 0.05) are highlighted with asterisks: * *P* < 0.05, ** *P* < 0.01, *** *P* < 0.001. (**c**) Correlation network between the differential gut microbiome and metabolome in the two groups. The networks were established by the Spearman correlation coefficient, with only features exhibiting moderate to strong correlations (*P* < 0.05, |Rho| > 0.2) included for presentation. The width of the connecting lines corresponds to the strength of the correlation, where yellow denotes a positive correlation and gray signifies a negative correlation.

We used MaAslin2 to elucidate the multivariate relationships between gut microbiota and clinical parameters. Our analysis exhibited a strong negative correlation between WOMAC scores and the abundance of *B. animalis* (Fig. 5a). This association indicates that Probio-M8 could play a part in symptom relief, as patients were instructed to avoid major dietary sources of *B. animalis*.

To explore the interplay between the fecal metabolome and KOA-related indices, we performed Spearman correlation analyses (*P* < 0.05, |Rho| > 0.15; Fig. 5b). The analysis identified several significant correlations: WOMAC scores exhibited strong positive correlations with prostaglandin E2, two saturated fatty acids (heptadecanoic acid and stearic acid), and two bile acids (chenodeoxycholic acid and cholic acid). Additionally, IFN-γ showed strong positive correlations with ethenodeoxyadenosine and heptadecanoic acid. Conversely, NAAD exhibited strong negative correlations with both WOMAC scores and IFN-γ.

To further explore the interplay between differential fecal microbiota and key metabolites in patients, we constructed a correlation network via Spearman’s rank correlation analysis (*P* < 0.05, |Rho| > 0.2; Fig. 5c). The analysis identified several notable correlations. *D. formicigenerans* exhibited positive correlations with xanthine, ethenodeoxyadenosine, heptadecanoic acid, isohyodeoxycholic acid, and chenodeoxycholic acid, while displaying a negative correlation with NAAD. Additionally, stearic acid was negatively correlated with *A. butyriciproducens* and *B. animalis*. Other significant correlations included a negative association between cholic acid and *R. hominis* and a positive association between testosterone and *B. stercoris*. These results shed light on the complex relationship among the gut microbiota, metabolites, and clinical parameters in patients with KOA.

## DISCUSSION

Probiotic adjuvant therapy has demonstrated clinical efficacy in mitigating a variety of medical conditions. Dysbiosis of the gut microbiota is now recognized as a primary modulator of host physiological processes, with the potential to influence disease development via alterations in the gut microbiota (9). Human health and host immunity are intricately connected to the gut microbiota. KOA is a multifactorial disease marked by excessive inflammation. The emerging concept of the gut-joint axis suggests a bidirectional interaction between the gut microbiota and joint health (39, 40). The current investigation explored the advantageous impacts of Probio-M8 when co-administered with chondroitin sulfate for KOA management. Our findings revealed associations between the gut microbiota, gut metabolome, and clinical parameters, thereby providing insights into the potential mechanisms by which probiotics may mitigate KOA.

The present study demonstrated that both chondroitin sulfate monotherapy and its combination with Probio-M8 resulted in significant reductions in WOMAC scores among individuals with KOA. However, the adjunctive use of Probio-M8 led to significantly lower WOMAC scores compared to those receiving conventional drugs alone. This finding is consistent with previous observations. For example, Lei reported that the consumption of probiotic-enriched skimmed milk significantly improved WOMAC scores and visual analog scale scores in individuals with KOA (22). Furthermore, the beneficial effects of Probio-M8 persisted for at least 1 month after discontinuation, suggesting a long-lasting impact of probiotic therapy. In addition to clinical symptom improvement, Probio-M8 treatment also led to reductions in the pro-inflammatory cytokine IFN-γ and increases in the anti-inflammatory cytokines IL-4 and IL-10 compared to placebo, further supporting the therapeutic potential of probiotics in modulating immune responses and preventing cartilage damage (41, 42).

Then, we employed microbial metagenomic sequencing coupled with comprehensive bioinformatics analysis to examine the gut microbiota in patients with KOA. Our findings of no significant difference in the Shannon index between the Probio-M8 and placebo groups at various time points, same as PCoA results, suggest that the observed symptom relief was not attributable to substantial alterations in gut microbiota diversity. We subsequently performed a detailed taxonomic analysis to assess the alternations in gut microbiota among the subjects. A significant rise was observed in the relative abundance of intestinal *B. animalis*, which exhibited a strong negative correlation with the WOMAC score. This underscores the pivotal role of Probio-M8 in symptom alleviation, particularly given that patients were instructed to avoid major dietary sources of *B. animalis*.

Our data indicate significant changes in the abundance of certain bacterial species in the gut microbiota of patients with KOA. Notably, several potentially beneficial and anti-inflammatory species, including *B. stercoris*, *A. butyriciproducens*, *R. hominis*, and *R. bromii*, exhibited significant increases in the Probio-M8 group compared to the placebo group, whereas *D. formicigenerans* decreased significantly. Other research has found a close association between specific gut bacteria and inflammation. For instance, *Bacteroides* are recognized for producing short-chain fatty acids that can inhibit inflammation (43). Similarly, *A. butyriciproducens*, *R. hominis*, and *R. bromii* are significant producers of butyrate, a signaling molecule that regulates intestinal homeostasis and has broad effects on the endocrine, nervous, and immune systems (40, 44). Conversely, *D. formicigenerans* has been implicated in distinguishing Takayasu arteritis patients from healthy controls, and it is linked to inflammation, gut permeability, and inflammatory bowel disorders (45, 46). These results suggest that the amelioration of KOA symptoms may be attributed to nuanced shifts in the gut microbiota.

Our results indicate that probiotic therapy as an adjuvant modifies the potential metabolic pathways of the gut microbiota. Specifically, the abundance of microbial metabolic pathways involved in 5-aminoimidazole ribonucleotide biosynthesis (PWY-6121, PWY-6122, and PWY-6277) was significantly increased by probiotic application. 5-Aminoimidazole ribonucleotide is a precursor to thiamine pyrophosphate, which acts as a cofactor for enzymes crucial in the production of antioxidants that counteract reactive oxygen species (ROS) (47, 48). Given the central role of ROS in the pathogenesis of KOA, influencing inflammatory responses and chondrocyte apoptosis to exacerbate cartilage damage, modulation of these pathways could have important implications for KOA management (49). We also observed significantly higher biosynthesis of cysteine, histidine, L-arginine, L-methionine, and L-glutamine (SULFATE-CYS-PWY, PRPP-PWY, PWY-7400, PWY-5345, and PWY-6549, respectively) in the Probio-M8 group. These amino acids show anti-inflammatory properties and can inhibit the activation of pro-inflammatory cytokines, thereby potentially reducing cartilage damage (50–53). For instance, cysteine is a key component of Wnt16, which has been shown to alleviate KOA and promote bone formation (54). The metabolic pathways affected by probiotics were primarily attributed to the majority of the gut bacteria, such as *E. coli*, *K. pneumoniae*, as well as differentially induced species like *A. hadrus* and *E. cloacae* complex.

Our findings demonstrate that probiotic intake significantly elevated predicted N-oleoylethanolamine concentrations. This bioactive lipid suppresses NF-κB pathway and attenuates TNF-α-induced inflammation via upregulation of CB2 and PPAR-α expression (55). Furthermore, our metagenome analysis also predicted a significant reduction in cholesterol and hypoxanthine levels in the Probio-M8 group compared to the placebo group. Feeding mice with a high-cholesterol diet elevated cholesterol levels, which can exacerbate cartilage damage and inflammatory responses, thereby worsening the severity of OA (56). Hypoxanthine, an intermediate product of purine metabolism, can generate free radicals during its metabolic process, leading to oxidative stress and cell damage (57). While these metabolites may influence KOA management, additional research is needed to clarify the underlying biochemical pathways and mechanisms.

Furthermore, we examined alterations in the fecal metabolome after the intake of Probio-M8. Notably, levels of prostaglandin E2 were significantly reduced in the fecal microbiome of subjects in the Probio-M8 group compared to the placebo group, even after discontinuation of the intervention. Prostaglandin E2 orchestrates OA pathogenesis by facilitating nociceptive signaling, disrupting cartilage homeostasis, and amplifying inflammatory cascades. Through EP4/TRPV1-dependent pathways, prostaglandin E2 sensitizes joint nociceptors and relays pain signals via dorsal root ganglia to higher somatosensory centers (58). Concurrently, EP4-mediated upregulation of chondrocyte MMP-13 suppresses proteoglycan synthesis and accelerates extracellular-matrix degradation (59). Prostaglandin E2 acutely activates mast cells via EP3 and chronically drives Th1/Th17/Th22-mediated inflammation via EP2 and EP4 (60). Moreover, the fecal levels of stearic acid, ethenodeoxyadenosine, and xanthine were significantly reduced in the Probio-M8 group. Stearic acid is often regarded as pro-inflammatory lipids that can activate inflammatory responses in chondrocytes through the lactate-HIF1α pathway (61). Ethenodeoxyadenosine, a product of DNA damage caused by lipid peroxidation due to chronic inflammatory processes, can induce oxidative stress and excessive ROS production (62). Xanthine can promote inflammation by stimulating the production of IL-1β (63). Conversely, Probio-M8 co-administration led to significant increases in the levels of testosterone and NAAD. Higher levels of testosterone have been linked to reduced pain in women undergoing knee joint surgery (64). In a cross-sectional analysis, low levels of testosterone have been significantly associated with the risk of OA (65). NAAD and L-glutamine can be converted into L-glutamic acid and NAD, which play crucial roles in maintaining extracellular matrix homeostasis and regulating chondrocyte metabolism (66). These metabolites exhibited strong correlations with certain gut microbial species that differed significantly between the two groups, including *A. butyriciproducens*, *B. stercoris*, *B. animalis*, *D. formicigenerans*, and *R. bromii*. Also, these metabolites were strongly correlated with levels of IL-10 and IFN-γ and WOMAC scores in KOA patients. These observations suggest that Probio-M8 consumption can alter the gut microbial composition and their functions, thereby regulating metabolites that inhibit chondrocyte oxidative apoptosis and ameliorate inflammatory conditions.

Our findings demonstrate that probiotic consumption decreased the ratio of lysoPCs to PCs, a potential biomarker for predicting KOA (67). The transformation of PCs into lysoPCs results in the release of arachidonic acid, a key precursor of prostaglandins, which are known to contribute to the inflammatory microenvironment of joint cartilage (68). However, no significant differences were detected between the Probio-M8 and placebo groups in serum levels of prostaglandin E2. Significantly lower fecal levels of prostaglandin E2 were observed in the Probio-M8 group. Furthermore, Probio-M8 co-administration reduced both fecal and circulating chenodeoxycholic acid and cholic acid. Elevated chenodeoxycholic acid, positively correlated with *D. formicigenerans*, bound to mitochondria to cause mitochondrial morphology damage, decreased mitochondrial membrane potential, and elevated mitochondrial calcium level, culminating in excessive ROS generation (69). Cholic acid levels were inversely associated with the beneficial bacterium *R. hominis*. Cholic acids precipitated stromal IL-33 release, subsequently activating ILC2s to secrete IL-5, thereby promoting tissue eosinophilia and inflammation (70). However, other studies have reported that both chenodeoxycholic acid and cholic acid exhibit significant anti-inflammatory and cartilage-protective effects in the pathogenesis of OA (71, 72).

**Fig 6:**
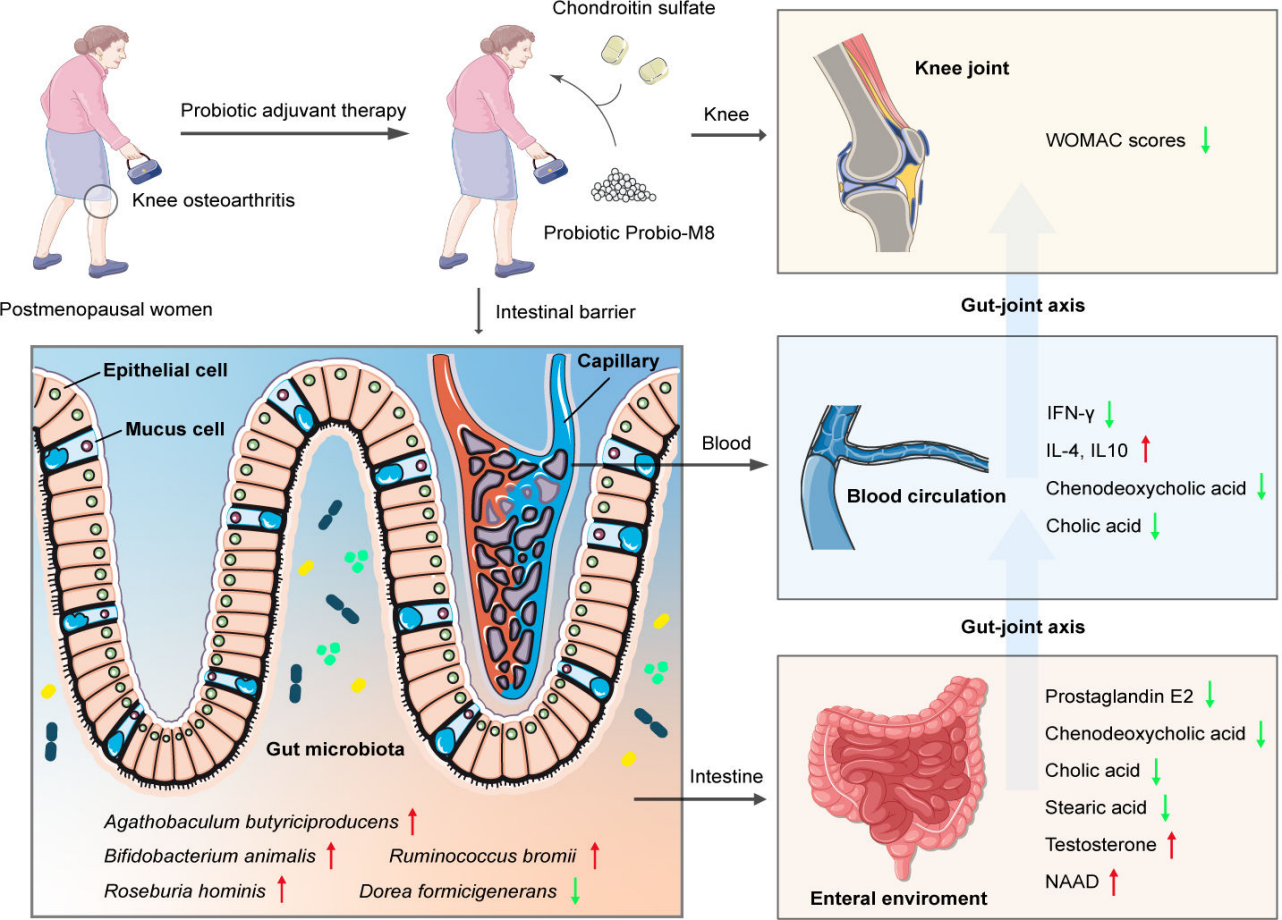
Schematic representation of probiotic-mediated pathways in KOA. This schematic diagram illustrates the key pathways by which Probio-M8 administration may modulate the gut-joint axis and influence the host response in patients with KOA. The red arrow signifies a probiotic-induced elevation in the levels of the indicated gut bacteria, parameters, or metabolites; conversely, the green arrow denotes their attenuation.

Our observations support that the gut microbiome modulation by Probio-M8 could potentially affect distal sites, although additional experimental evidence is required to validate this hypothesis(Fig. 6). The restricted sample size limits statistical power and external validity. Systemic estradiol concentrations and other related confounders such as diet, obesity, and genetic predisposition were not prospectively quantified in the present cohort, precluding complete assessment for their potential residual influence on gut microbiota of KOA patients. Consequently, the conclusions need to be interpreted with circumspection.

### Conclusion

In summary, the current investigation revealed that the co-administration of Probio-M8 with conventional therapy effectively alleviated symptoms of KOA. This synergistic approach modulated the gut microbiota and its metabolic functions, thereby directly influencing the gut-joint axis. Specifically, the intervention resulted in substantial changes in the abundance of anti-/pro-inflammatory microbial species, gut bioactive metabolites, serum bile acids, and related metabolic pathways. Our findings highlight the potential of Probio-M8 to expand the treatment options for KOA by targeting the gut-joint axis. The observed health-promoting effects suggest that probiotics may serve as a valuable adjunct to conventional therapies, offering a novel strategy for managing related chronic disorders. Future research should delve deeper into the mechanisms behind these effects and investigate the long-term clinical significance of probiotic interventions in KOA management.

## Data Availability

The raw sequence data reported in this paper have been deposited in the Genome Sequence Archive (Genomics, Proteomics & Bioinformatics 2021) in National Genomics Data Center (Nucleic Acids Res 2022), China National Center for Bioinformation/Beijing Institute of Genomics, Chinese Academy of Sciences (GSA: CRA015191) that are publicly accessible at https://ngdc.cncb.ac.cn/gsa.

## SUPPLEMENTAL MATERIAL

Supplemental material msystems.00862-25-s0001.docx Fig. S1 and S2; Tables S1 and S2.

ASM does not own the copyrights to Supplemental Material that may be linked to, or accessed through, an article. The authors have granted ASM a non-exclusive, world-wide license to publish the Supplemental Material files. Please contact the corresponding author directly for reuse.
