# Supplementary material for: Bifidobacterium animalis subsp. lactis Probio-M8 enhances chondroitin efficacy for knee osteoarthritis in postmenopausal women via the gut-joint axis
Source: mSystems. 2025 Nov 28;10(12):e00862-25. doi: 10.1128/msystems.00862-25 (PMC12710372; doi:10.1128/msystems.00862-25)
Supplement: Supplemental material — Fig. S1 and S2; Tables S1 and S2. [file msystems.00862-25-s0001.docx]

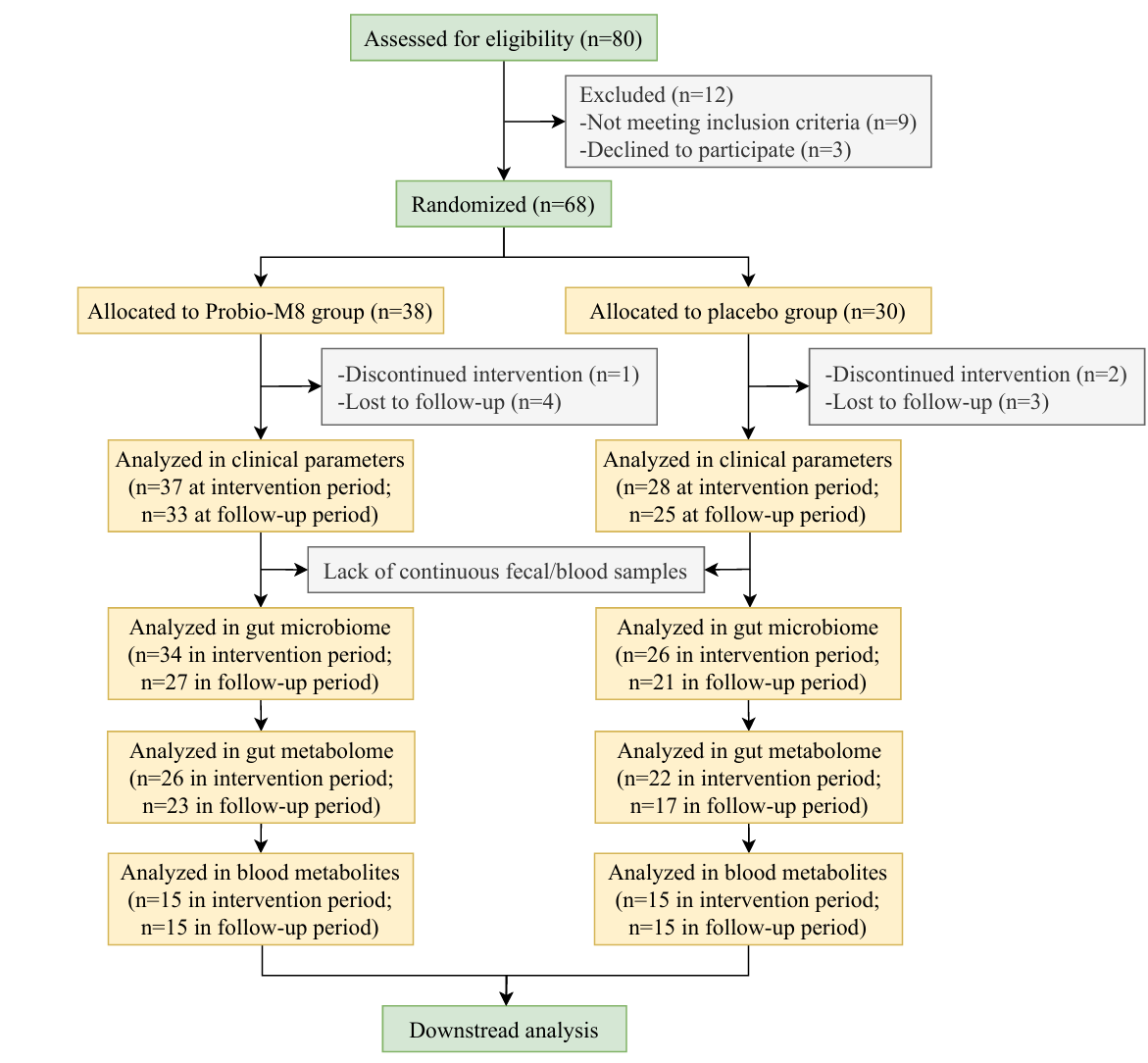


Figure S1. Screening and sample collection. A total of 68 patients were recruited following screening based on predefined inclusion and exclusion criteria. Randomization of participants was conducted using R and a random number generator to ensure balanced group allocation. Throughout the study, no adverse events or serious adverse events were reported by any patient. At each time point, compliance and clinical presentations were jointly assessed by a team of physicians and scientists to ensure the accuracy and integrity of the data.


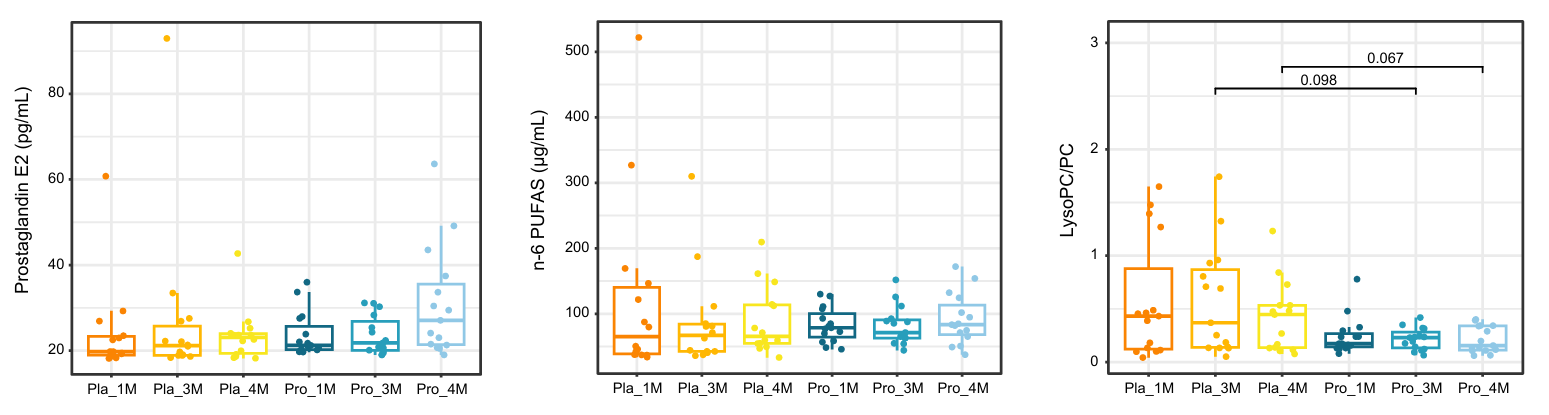


Figure S2. Levels of serum prostaglandin E2, omega-6 polyunsaturated fatty acids (n-6 PUFAs), and the ratio of lysophosphatidylcholine to phosphatidylcholine (lysoPC/PC) at months 1, 3, and 4. The units for lysoPC and PC are μmol/L and pg/mL, respectively. Statistical differences in levels between subgroups were evaluated using the Wilcoxon test, with *P*-values < 0.1 indicated.

Table S1 Diagnostic criteria for knee osteoarthritis.

| Number | Symptoms |
| --- | --- |
| 1 | Knee pain on most days of the prior month |
| 2 | Joint-space narrowing, subchondral bone sclerosis or cystic change, and marginal osteophyte formation |
| 3 | Age ≥ 50 years |
| 4 | Morning stiffness ≤ 30 minutes |
| 5 | Crepitus on motion |

Note: Diagnosis is established when criterion 1 is satisfied in conjunction with any two of criteria 2–5.

Table S2. Baseline demographics and clinical characteristics of participants in the two groups.

| Parameters | Probio-M8 group (n=37) | Placebo group (n=28) | *P*-value |
| --- | --- | --- | --- |
| Age | 61.57±6.94 | 62.79±5.56 | 0.44 |
| WOMAC scores | 45.76±2.93 | 46.64±2.08 | 0.16 |
| IL-2 (pg/ml) | 4.64±0.54 | 4.76±0.54 | 0.41 |
| IL-4 (pg/ml) | 2.81±0.57 | 2.96±0.77 | 0.38 |
| IL-6 (pg/ml) | 6.94±2.42 | 7.71±5.54 | 0.50 |
| IL-10 (pg/ml) | 4.26±1.41 | 4.23±0.54 | 0.90 |
| IL-17A (pg/ml) | 6.46±2.40 | 7.50±2.22 | 0.08 |
| TNF-α (pg/ml) | 2.40±0.78 | 2.41±0.59 | 0.93 |
| IFN-γ (pg/ml) | 4.18±0.67 | 4.38±0.49 | 0.16 |

Note: Data are presented as mean ± standard deviation. Statistical differences in levels between Probio-M8 and placebo groups were evaluated using the Wilcoxon test.
